# Early IL-1 Signaling Promotes iBALT Induction after Influenza Virus Infection

**DOI:** 10.3389/fimmu.2016.00312

**Published:** 2016-08-16

**Authors:** Katrijn Neyt, Corine H. GeurtsvanKessel, Kim Deswarte, Hamida Hammad, Bart N. Lambrecht

**Affiliations:** ^1^Laboratory of Immunoregulation, VIB Inflammation Research Center, Ghent, Belgium; ^2^Department of Respiratory Medicine, Ghent University, Ghent, Belgium; ^3^Department of Viroscience, Erasmus MC, Rotterdam, Netherlands; ^4^Department of Pulmonary Medicine, Erasmus MC, Rotterdam, Netherlands

**Keywords:** influenza, innate immunity, TLO, IL-1, iBALT, CXCL13

## Abstract

Inducible bronchus-associated lymphoid tissue (iBALT) is a long lasting tertiary lymphoid tissue that can be induced following influenza A virus (IAV) infection. Previous studies have shown that iBALT structures containing germinal center (GC) B cells protect against repeated infection by contributing locally to the cellular and humoral immune response. If we are to exploit this in vaccination strategies, we need a better understanding on how iBALT structures are induced. One hypothesis is that the strength of the initial innate response dictates induction of iBALT. In the present study, we investigated the role of interleukin (IL)-1 and IL-1R signaling on iBALT formation. Mice lacking the IL-1R had a delayed viral clearance and, thus, a prolonged exposure to viral replication, leading to increased disease severity, compared to wild-type mice. Contradictorily, iBALT formation following clearance of the virus was heavily compromised in *Il1r1*^−/−^ mice. Quantification of gene induction after IAV infection demonstrated induction of IL-1α and to a much lesser extent of IL-1β. Administration of recombinant IL-1α to the lungs of wild-type mice, early but not late, after IAV infection led to more pronounced iBALT formation and an increased amount of GC B cells in the lungs. Bone marrow chimeric mice identified the stromal compartment as the crucial IL-1 responsive cell for iBALT induction. Mechanistically, Q-PCR analysis of lung homogenates revealed a strongly diminished production of CXCL13, a B cell-attracting chemokine, in *Il1r*^−/−^ mice during the early innate phase of IAV infection. These experiments demonstrate that appropriate innate IL-1α–IL-1R signaling is necessary for IAV clearance and at the same time instructs the formation of organized tertiary lymphoid tissues through induction of CXCL13 early after infection. These findings are discussed in the light of recent insights on the pathogenesis of tertiary lymphoid organ formation in the lung in various diseases where the IL-1 axis is hyperactive, such as rheumatoid arthritis and COPD.

## Introduction

Influenza A virus (IAV) is a respiratory pathogen that causes seasonal or pandemic outbreaks with severe outcome in elderly and immune compromised patients. Epithelial cells are the first target cells for IAV infection (1, 2) and also coordinate the innate immune defense to prevent spreading of the virus, *via* production of type I interferons (IFNs). Interleukin (IL)-1α and IL-1β are among the first cytokines that are secreted by epithelial cells and macrophages at sites of IAV replication (3). Secretion of IL-1β requires activation of the Nlrp3 inflammasome that leads to activation of caspase-1 and cleavage of pro-IL-1β into IL-1β. Infection with IAV leads to activation of the Nlrp3 inflammasome in a process requiring the type I IFN-induced RNAse L/OAS system, while the virus actively suppresses IL-1β production and Nlrp3 activation *via* the NS1 protein (4–6). IL-1 induces the expression of endothelial adhesion molecules that promote the entry of innate inflammatory cells like neutrophils, NK cells, dendritic cells (DCs), and monocytes resulting in a double effect on the host. On the one hand, it promotes survival by killing virus-infected cells, clearing debris, and alarming the adaptive immune response. On the other hand, overzealous neutrophil recruitment can also cause inflammatory pathology that can ultimately lead to diffuse alveolar damage and death (6–10). Not surprisingly, the outcome of genetic deficiency of key components in IL-1 generation or signaling has been very different depending on the severity of the IAV infection (2, 9, 11).

Simultaneously with the activation of the innate immune response, adaptive immune responses are initiated in the draining lymph nodes by antigen-presenting migratory DCs. The architecture of lymph nodes promotes contact between antigen-presenting DCs and rare antigen-specific T cells and B cells of the adaptive immune system to maximize the immune response against a certain antigen. Antigen-specific T lymphocytes undergo clonal proliferation upon encounter with antigen presented by antigen-presenting cells and migrate back to the site of inflammation as T effector memory (T_EM_) cells or become central memory T cells (T_CM_) or T resident memory (T_RM_) cells (12). Antibody production is initiated from B lymphocytes that differentiate into plasmablasts, immediately, or become plasma cells after going through a germinal center (GC) reaction that promotes somatic hypermutation and affinity maturation of B cells (13).

The coordinated events of T and B cell activation induced by virus-laden DCs mainly occur in secondary lymphoid organs (SLOs) like lymph nodes and spleen that develop during embryogenesis at predefined areas, often at the crossroads of lymphatic vessels (14). However, highly organized structures of T and B cells can also be formed in the lung after birth as an adaptation to the increased demand for a localized immune response. Various names, such as lymphoid tissue neogenesis, ectopic lymphoid tissue, and tertiary lymphoid structures have been used to describe these structures. Furthermore, they are often named after the anatomical region in which they occur. In the lungs, for example, lymphoid aggregations can often be found in close proximity to bronchi, and hence, these are called inducible bronchus-associated lymphoid tissues (iBALT). As they resemble SLOs anatomically and functionally, yet, only develop after birth as a result of chronic immune stimulation, they can also be called tertiary lymphoid organs (TLOs), even when found within the boundaries of another organ.

Tertiary lymphoid organs have been implicated in protection against IAV. Mice that lack SLOs can mount a rapid CD8 T cell response during IAV infection due to the induction iBALT (15), induced after clearance of IAV infection. Such TLO structures are generally formed in close proximity to bronchi after IAV infection, but can also be observed in the lung interstitium and are fully formed 17 dpi. iBALT structures that are induced in mice with functional lymph nodes can serve as an additional priming site for T cells (16) and can also contribute to the humoral immune responses (17). Once formed, iBALT structures can mount high affinity immune responses to other antigenic stimuli than the initiating stimulus due to the presence of GCs that allow somatic hypermutation and affinity maturation, complementing the immune response in the draining lymph nodes (16, 18). Furthermore, iBALT structures could be the perfect environment for depots of viral antigen that were described long after viral clearance and recovery from viral infection and possibly related to the induction or maintenance of virus-specific T_RM_ cells (19, 20).

Despite morphological and functional similarities between SLOs and TLOs, the pathways that control formation and maintenance of TLOs are less clear. Generally, the molecular pathway that organizes T and B cells in discrete areas resembles the highly regulated inductive pathway for SLO development. Production of CCL19, CCL21, CXCL12, and CXCL13 by stromal cells and B cells helps to organize and retain T and B cells in discrete areas (21–26). Also IL-7 seems required for TLO formation, e.g., in joints of rheumatoid arthritis patients and mouse models (27–30) and lungs of idiopathic pulmonary hypertension (IPAH) patients (31). It is more controversial which cells give the initial instruction for stromal cells to produce these chemokines. During SLO development in the fetal period, this is the distinct task of lymphoid tissue inducer (LT_i_) cells, a cell type that develops from Flt3^+^ and Flt3^−^ α_4_β_7_ integrin-positive common progenitors that also forms innate lymphoid cells (ILC), and get expanded in response to Flt3L injections (32, 33). During formation of the lymph nodes and spleen, LT_i_ cells provide signals for lymphoid organogenesis like lymphotoxin-beta (LTβ) acting on the LTβR on stromal cells, but the precise signals might differ from organ to organ. *Flt3l*^−/−^ mice for example have reduced LT_i_ cells and lack Peyer’s patches, but still have lymph nodes (33). However, research in *Id2*^−/−^ and *Rorc*^−/−^ mice, which lack LT_i_ cells, showed that LT_i_ cells were dispensable for the initial TLO induction after IAV infection or other forms of TLO induction (15, 34–38). Although LT_i_ cells seem not strictly necessary for TLO induction, an instructive LTβ–LTβR signal remains essential for proper TLO development (25, 39). B cells, T cells, and DCs are heavily induced during inflammatory processes and all express LTα1β2 on their cell surface (17, 35, 40, 41); therefore, they are perfect candidates to function as a substitute for LT_i_ cells.

Whatever the precise molecular mechanisms of TLO induction might be, these TLO structures are virtually always seen at sites of inflammation. Yet, which inflammatory cytokines contribute to TLO induction is currently unknown. After many insults to the lung, including viral or bacterial infection, IL-1α and IL-1β are among the first cytokines to be secreted (3). IL-1 secretion induces the expression of endothelial adhesion molecules that promote the entry of innate and adaptive immune cells and could, thus, promote TLO formation. On the other hand, it is also known that IL-1R^−/−^ mice have a delayed viral clearance and, thus, a longer exposure to viral particles (7). Chronic immune stimulation is often assumed to lead to TLO formation (42). If IL-1 limits viral replication, it could reduce the trigger for TLO induction.

In this paper, we addressed the role of IL-1 and IL-1R in TLO formation in the lung. We show that the iBALT-inducing events are initiated early after infection, long before the virus is cleared. More specifically, we show that early IL-1R signaling is necessary for proper IAV-associated iBALT and GC induction and that prolonged viral presence does not automatically lead to iBALT induction.

## Materials and Methods

### Ethics Statement

All experiments were approved by the independent animal ethics committee “Ethische Commissie Proefdieren – faculteit Wetenschappen Universiteit Gent en VIB-site Ardoyen” (identification number: EC 2013_070). Animal care and used protocols adhere to the Belgian Royal Degree of 29th May, 2013 for protection of experimental animals. European guideline 2010/63/EU is incorporated in this Belgian legislation.

### Mice

C57Bl/6 mice (8–10 weeks) were purchased from Harlan Laboratories. IL-1R^−/−^ mice were bred in-house and housed in specific pathogen-free housing.

To create chimeric mice, IL-1R^−/−^ or wild-type acceptor mice were irradiated sublethally (9 Gy) and reconstituted with 2 × 10^6^ bone marrow cells i.v. from wild-type or IL-1R^−/−^ donor mice respectively 4 h after irradiation. Mice were used for experiment at least 10 weeks after reconstitution.

### Influenza Virus Infection

Mice were infected intranasally with 10^5^ TCID50 H3N2 influenza virus X-31 (Medical Research Council) or mock (allantoic fluid of uninfected eggs); diluted in 50 μl PBS. Weight loss was monitored daily. For suppletion assays, mice were treated intratracheally with 80 μg carrier-free recombinant IL-1α (R&D) at 2 or 10 dpi.

### TCID50 Assay Viral Titers

Lungs were homogenized with a tissue homogenizer in 1 ml PBS and centrifuged (5 min, 400 *g*) to remove cellular debris before storage at −80°C. Titers of infectious virus were determined in triplicate by titration on MDCK cells in serum-free TPCK-treated trypsin-containing medium. Viral titers were determined by measuring chicken red blood cell agglutination activity in the cell supernatant after 7 days of infection of MDCK cells by using the calculation method of Reed and Muench.

### Isolation of Lung Cells

Mice were sacrificed and the lungs were removed. Single-cell suspensions were prepared by digestion in collagenase/DNase solution for 30 min at 37°C. After digestion, the suspension was filtered over a 100 μm filter and red blood cells were lysed with osmotic lysis buffer (10 mM KHCO_3_, 155 mM NH_4_Cl, 0.1 mM EDTA in ddH_2_O).

### Flow Cytometry

Lung cells were stained extracelullarly with anti-CD3 (17A2, conjugated to AF700, eBioscience), anti-CD19 (1D3, conjugated to APC, BD Bioscience), anti-IgM (R6-60.2, conjugated to PerCp-Cy5.5, BD Bioscience), anti-IgD (11-26c.2a, conjugated to PE, BD Bioscience), anti-CD95 (Jo2, conjugated to PE-Cy7, BD Bioscience), anti-GL7 (GL7, conjugated to Fitc, BD Bioscience), and a fixable live-dead marker (conjugated to eFl506, eBioscience).

Acquisition of samples was performed on a LSR II or Fortessa cytometer equipped with FACSDiva software (BD biosciences). GC B cells were defined as CD3^−^CD19^+^IgM^−^IgD^−^CD95^+^GL7^+^. Final analysis and graphical output were performed using FlowJo software v9 (Tree Star, Inc.).

### Real-time Quantitative RT-PCR

Quantitative RT-PCR for IL-1α, IL-1β, LTβ, CCL19, CCL21, CXCL12, and CXCL13 were performed on RNA obtained from whole lung homogenates. Total RNA was extracted using Tripure reagent (Roche) according to the manufacturer’s protocol. RNA was resuspended in diethyl-polycarbonate (DEPC, Sigma)-treated water. A total of 1 μg RNA was used for reverse transcription using the Transcriptor High Fidelity Reverse Transcriptase kit (Roche) according to the manufacturer’s protocol. The subsequent target amplification on triplicates of each cDNA sample was performed using the Universal Probe Library system from Roche, which contains fluorescent hydrolysis probes of eight locked nucleic acids (LNA). Primers were designed with the help of the web-based application Probefinder (https://qpcr.probefinder.com), and a minimum of two primer pairs per target were analyzed. Primers were validated first using the LC480 SybrGreenI Master (Roche) with melting curve analysis (TM calling) in the LC480 Software and then using the LC480 Probes Master. Aspecific primer pairs were discarded. Table 1 shows a comprehensive view of the primer/probe combinations chosen. PCR conditions were: 5′ pre-incubation at 95°C followed by 45 amplification cycles of 10″ at 95°C, 10″ at 60°C, and 20″ at 72°C using a Lightcycler 480 (Roche). PCR amplifications for the housekeeping genes encoding *Hprt* or *L27* were performed during each run for each sample to allow normalization between samples.

**Table 1:** **Q-PCR primers**.

Target	Ensemble transcript	Fwd primer	Rvs primer	Probe #
CXCL12	ENSMUSG00000061353	GGTTCTTCGAGAGCCACATC	TTCTTCAGCCGTGCAACA	21
CXCL13	ENSMUSG00000023078	CAGAATGAGGCTCAGCACAG	ATGGGCTTCCAGAATACCG	80
CCL19	ENSMUSG00000071005	GGTGCCTGCTGTTGTGTTC	CTGGTGCTGTTGCCTTTGT	29
CCL21	ENSMUSG00000094121	ACCCAAGGCAGTGATGGA	GCTTCCGGGGTAAGAACAG	74
IL-1α	ENSMUSG00000027399	TTGGTTAAATGACCTGCAACA	GAGCGCTCACGAACAGTTG	52
IL-1β	ENSMUSG00000027398	AAAGCTTGGTGATGTCTGGTC	AAAGGACATGGAGAACACCACT	10

### Histology

Lungs were inflated with 1 ml 1:1 PBS-OCT (Tissue Tec), snap frozen in liquid nitrogen and stored at −80°C. Frozen 8 μm sections were fixed in 4% PFA and blocked in a 1% blocking buffer (Roche).

Immunofluorescence staining was performed by staining for B220 (RA3-6B2, Rat-a-mB220 conjugated to PE, BD Bioscience + Goat-a-Rat conjugated to AF555, Invitrogen), CD4 (RM4-5, Rat-a-CD4 conjugated to Fitc, eBioscience + Rab-a-Fitc conjugated to AF488, Invitrogen), CD8 (53-6.7, Rat-a-CD8 conjugated to Fitc, BD bioscience + Rab-a-Fitc conjugated to AF488, Invitrogen), CD11c (N418, Hamster-a-mCD11c conjugated to AF647, eBioscience), and GL7 (GL7, Rat-a-mGL7 Fitc, BD bioscience + a-Fitc conjugated to HRP, Jackson + a-HRP conjugated to Fitc, Jackson). Where necessary, slides were incubated with 10% normal rat serum to prevent aspecific binding of antibodies. Slides were counterstained with Dapi and digitized on a LSM710 microscope (Zeiss). All depicted pictures are representative of at least five mice per group. Images were analyzed using Imaris software (Bitplane).

### Statistical Analysis

All experiments were performed using three to six animals per group. All experiments were performed at least two times. The difference between groups was calculated using the Student’s *t* test for unpaired data (Prism version 6; GraphPad Software, Inc.). Data are depicted as mean ± SEM. Differences were considered significant when *p* < 0.05.

Analysis of the repeated relative body weight data was performed using the residual maximum likelihood (REML) as implemented in Genstat v17 (43). The following linear mixed model (random terms underlined) was fitted to the data: *Y*_ijkt_ = μ + genotype_j_ + treatment_k_ + time_t_ + (genotype⋅treatment)_jk_ + (genotype⋅time)_jt_ + (treatment⋅time)_kt_ + (genotype⋅treatment⋅time)_jkt_ + (mouse⋅time)_it_ + *residual*_ijkt_, where *Y*_ijkt_ is the relative body weight of i-th mouse having genotype j, k-treated, and measured at time point *t* (*t* = 3–17 days; unequally spaced) and μ is the overall mean calculated for all mice considered across all time points. A first order antedependence covariance structure was used to model the within-subject correlation. The significance of the comparison between WT-X31 and KO-X31 across time was assessed by an *F*-test.

A log-linear model (Poisson distribution and log link) was fitted to the amount of viral particles measured by hemagglutination inhibiton assay. The dispersion parameter was set as free. Significance of main effects GENOTYPE and TIME and the GENOTYPE⋅TIME interaction effect was assessed by an *F*-test.

## Results

### *Il1r1*^−/−^ Mice Have Prolonged Viral Load but Are Unable to Induce iBALT

**Figure 1:**
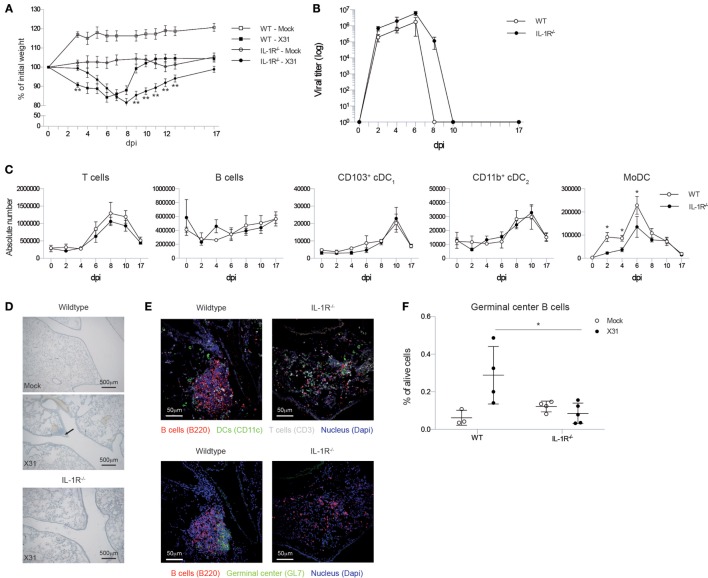
***Il1r1*^−/−^ mice have a prolonged viral load in the lungs, but do not develop iBALT structures**. **(A)** Weight loss curve as percentage of the initial bodyweight for wild-type (squares) and *Il1r1*−/− mice (circles) that have been infected with mock (white) or X31 (black) virus. The significance of the comparison between WT-X31 and KO-X31 across time was assessed by an *F*-test (*F* < 0.001), differences between individual time points were assessed by a *t* test. **(B)** Viral titers in the lung of wild-type (white) or *Il1r1*^−/−^ (black) mice determined by hemagglutination-inhibition assay after culture with MDCK cells. Significance of main effects GENOTYPE and TIME and the GENOTYPE⋅TIME interaction effect was assessed by an *F*-test (*F* = 0.022; *F* < 0.01; *F* = 0.984, respectively). **(C)** Numbers of T and B cells, CD103^+^ cDC_1_ and CD11b^+^ cDC_2_ DCs, and monocyte-derived cells (MoDC) in the lungs of wild-type (white) and *Il1r1*^−/−^ mice (black). **(D)** Hematoxylin-stained lung sections 17 dpi of wild-type and *Il1r1*^−/−^ mice. Images are representative for at least three mice per group. **(E)** Confocal images of lung sections of wild-type and *Il1r1*^−/−^ mice at 17 dpi. Sections were stained with either B220 (red), CD11c (Green), CD3 (Gray) and dapi (blue) or B220 (red), GL7 (Green), and dapi (Blue). Shown images are representative for five mice per group. **(F)** Proportion of germinal center B cells (GL7^+^) in the lungs of wild-type and *Il1r1*^−/−^ mice infected with mock (white) or X31 (black) virus at 17 dpi determined by flow cytometry. All experiments were performed at least twice with 4–6 mice per group. *p < 0.05; **p < 0.01.

To assess the immune response to IAV infection in mice lacking signaling *via* IL-1R, we infected *Il1r1*^−/−^ mice and monitored weight loss and viral load in the lungs. Wild-type mice showed maximum weight loss around 6 dpi, and bodyweight was fully restored around 10 dpi. *Il1r1*^−/−^ mice lost weight with slower and prolonged kinetics, and reaching a nadir at 8 dpi. Like wild-type mice they did manage to gain weight again, but did not recover to their starting bodyweight before 17 dpi (Figure 1A). This difference in the weight loss curve was also reflected in the viral load in the lungs (Figure 1B). Viral load in wild-type mice peaked around 6 dpi, yet was cleared at 8 dpi. *Il1r1*^−/−^ mice had systematically higher viral loads during the entire course of infection and did not clear the infection completely at 8 dpi as an estimated remaining titer of 100,000 viral particles is detected at this time point.

Viral clearance depends on induction of adaptive immunity by DCs that activate CD8 and CD4 T cells and a humoral immune response by B cells. Total numbers and kinetics of increase of T and B cells and conventional DCs were not altered in *Il1r1*^−/−^ mice in response to IAV infection. Lung conventional DCs consist of various subsets that have different functions and can be discriminated based on cell surface markers CD11b and CD103 (44, 45). As soon as there is inflammation in the lung, monocytes can also be recruited and these can rapidly differentiate into a MHCII^+^CD11c^+^ cell type (so called monocyte-derived DC, MoDC) that also expresses the macrophage marker CD64 (46). In contrast to the conventional CD103^+^ and CD11b^+^ DCs, the accumulation of CD11c^+^CD64^+^ MoDCs was reduced in the lungs of *Il1r1*^−/−^ mice (Figure 1C).

To evaluate the effect of the higher and prolonged viral exposure on IAV-associated iBALT formation, we visualized iBALT structures in the lungs by hematoxilin staining. Generally, clusters of cells near the bronchi were more readily detected in wild-type mice than in *Il1r1*^−/−^ mice (Figure 1D). Because a hematoxilin stain did not allow us to evaluate if the inflammatory clusters of cells were organized and immunologically active iBALT structures, we stained frozen lung sections for B cells, T cells, DCs, and GC B cells and analyzed them by confocal microscopy. In wild-type mice, we could easily detect organized structures composed of B cells, T cells, and DCs and B cell aggregates that contain GC B cells, but we were unable to detect similar infiltrates in *Il1r1*^−/−^ mice (Figure 1E). To quantify the presence or absence of iBALT structures we measured the proportion of GC B cells in the lungs at 17 dpi by flow cytometry as a measure for biologically active iBALT. As GL7^+^ GC B cells are not found in the lungs of mice in the absence of iBALT, we believe this is a good approximation of the amount of iBALT formed (17). Wild-type mice showed an induction of GC B cells upon IAV infection, but this induction was absent in *Il1r1*^−/−^ mice (Figure 1F). Taken together, these data suggest that although *Il1r1*^−/−^ mice had a higher and prolonged exposure to viral particles in the lung, they were unable to form organized iBALT structures in the lung, pointing to an essential role for IL-1 cytokines in TLO induction following influenza virus infection.

### IL-1α Administration Promotes Induction of iBALT Structures in the Lung

**Figure 2:**
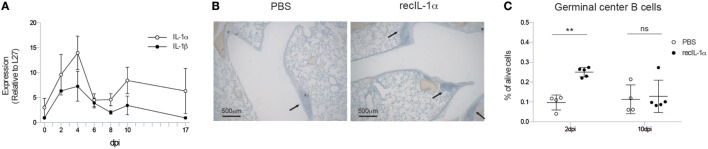
**Early IL-1α signaling is sufficient and necessary for iBALT induction**. **(A)** mRNA expression of IL-1α (white) and IL-1β (black) relative to the housekeeping gene L27 in the lungs of wild-type mice. **(B)** Hematoxylin-stained lung sections 17 dpi of mice treated with PBS or recIL-1α 2dpi. Images are representative for at least three mice per group. **(C)** Proportion of germinal center B cells 17 dpi in the lungs of wild-type mice treated with PBS (white) or recombinant IL-1α (black) i.t. 2 or 10 dpi. All experiments were performed at least twice with 3–5 mice per group. ns, not significant. **p < 0.01.

As the mere presence of viral particles was not enough to trigger the iBALT initiation and IL-1R signaling was necessary, we quantified expression of IL-1α and IL-1β, which signal both *via* IL-1R, in the lungs after IAV infection. Both cytokines were induced after infection in a bimodal curve with a first peak around 4 dpi and a second, but smaller, peak around 10 dpi (Figure 2A). In general, the induction of mRNA for IL-1α was more pronounced compared with IL-1β.

We, next, addressed if administration of IL-1 cytokine would be enough to further boost iBALT induction in IAV-infected mice. Since IL-1α and IL-1β have similar effects on the IL-1R, and as induction of IL-1α was more pronounced after IAV infection, we chose to only administer IL-1α. When recombinant IL-1α was administered i.t. 2 days post IAV infection in wild-type mice, clustering of inflammatory cells could be detected more readily on lung sections compared to PBS-treated IAV-infected mice (Figure 2B). To quantify biologically active iBALT, we again quantified the proportion of GC B cells by flow cytometry. Administration of recombinant IL-1α at 2 dpi resulted in a higher proportion of GC B cells in the lungs 17 dpi compared to PBS administration (Figure 2C). However, when the administration of recombinant IL-1α was only initiated at 10 dpi, no differences in the proportion of GC B cells could be observed between IL-1α and PBS-treated groups (Figure 2C). This suggests that early, but not late, IL-1R signaling is necessary and sufficient to promote GC B cell positive iBALT structures.

### IL-1R Signaling on Stromal Cells Is Necessary to Induce GC B Cells

**Figure 3:**
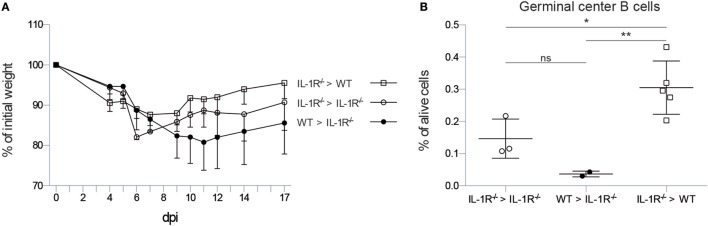
**IL-1R signaling on stromal cells is necessary to induce germinal center B cells in the lung**. **(A)** Weight loss curve after infection with X31 virus as percentage of the initial bodyweight for wild-type (squares) and IL-1R^−/−^ (circles) mice reconstituted with wild-type (black) or IL-1R^−/−^ bone marrow (white). **(B)** Proportion of germinal center B cells 17 dpi in the lungs of wild-type (squares) and IL-1R^−/−^ (circles) mice reconstituted with wild-type (black) or IL-1R^−/−^ bone marrow (white). ns, not significant. *p < 0.05. **p < 0.01.

As early IL-1R signaling is necessary to induce iBALT structures in the lung, we sought to identify the cell type that is responsive to IL-1 signals. Therefore, we constructed bone marrow chimeric mice in which either the radiosensitive hematopoietic or the radioresistant stromal compartment was deficient for *Il1r1*. As a control, we also reconstituted *Il1r1*^−/−^ mice with *Il1r1*^−/−^ bone marrow as a substitute for intact *Il1r1*^−/−^ mice and control for irradiation effects. *Il1r1*^−/−^ mice that received wild-type bone marrow followed a weight loss curve characterized by a longer weight loss and slower recovery, as observed in *Il1r1*^−/−^ mice. In contrast, wild-type mice that were reconstituted with *Il1r1*^−/−^ bone marrow cells followed a weight loss curve that resembled the one observed in wild-type mice with a maximum of approximately 15% weight loss. Surprisingly, *Il1r1*^−/−^ mice that were reconstituted with IL-1R sufficient bone marrow had a tendency to lose more weight than the *Il1r1*^−/−^ mice that were reconstituted with IL-1R-deficient bone marrow (Figure 3A). We also counted the proportion of GC B cells in the lung. Wild-type mice with IL-1R-deficient hematopoietic cells were able to induce a higher proportion of GC B cells than *Il1r1*^−/−^ mice with normal hematopoietic cells (Figure 3B), suggesting that IL-1 boosts the formation of iBALT structures by signaling to radioresistant stromal cells.

### CXCL13 Expression Is Reduced in *Il1r1*^−/−^ Mice

**Figure 4:**
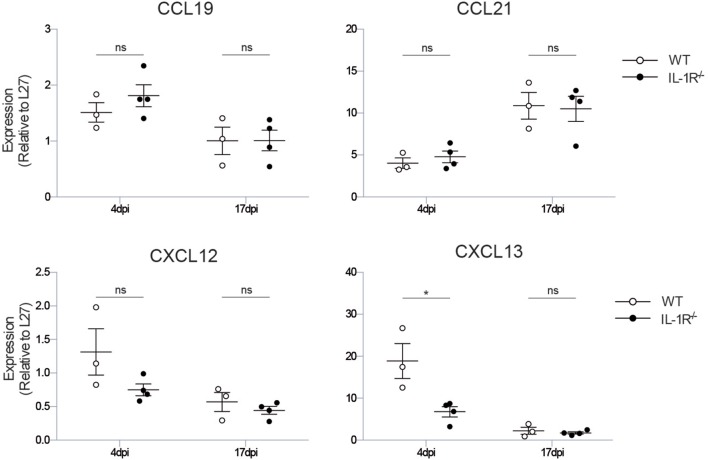
***Il1r1*^−/−^ mice have a defect in the production of the iBALT-inducing chemokine CXCL13**. mRNA expression levels of the chemokines involved in iBALT induction: CXCL12, CXCL13, CCL19, CCL21 relative to the expression of housekeeping gene L27. The experiment was performed twice with three to five mice per group. ns, not significant. *p < 0.05.

To define which downstream iBALT-instructive signals are induced by IL-1R signaling in radioresistant cells, we measured the expression level of the chemokines CXCL12, CXCL13, CCL19, and CCL21, which all have been implicated in iBALT formation. The chemokines CCL19 and CCL21 instruct the organization of T cell zones in iBALT structures, but were not impaired in *Il1r1*^−/−^ mice (Figure 4). The chemokine CXCL12 is important for B cell lymphopoiesis, but expression of this chemokine was only slightly reduced early after infection in *Il1r1*^−/−^ mice. The chemokine CXCL13 functions as an LT_i_ and B cell chemoattractant, and its expression was reduced 4 dpi in *Il1r1*^−/−^ mice (Figure 4). None of the chemokines involved in iBALT formation and organization was differentially expressed at 17 dpi, when iBALT had fully developed in wild-type mice, showing that the instructive chemokine signals are given early after infection when the virus is not yet cleared from the lungs. Although CXCL13 expression was impaired early after infection, the total amount of B cells in the lungs was not significantly altered at the time iBALT was present (Figure 1C), suggesting that it is not recruitment of B cells to the lungs that is impaired in *Il1r1*^−/−^ mice but that the B cells in the lung fail to cluster into organized iBALT structures. Whether this is a direct or indirect effect of defective IL-1R signaling on stromal cells remains a subject for future experiments.

## Discussion and Review of the Literature

The IL-1 axis has been described previously to be responsible for inflammatory pathology in the lung, resulting in increased mortality, increased viral titers, and neutrophil recruitment following IAV infection (7). *Il1r1*^−/−^ mice indeed suffered more from the mild X31 IAV infection and displayed a tendency to higher viral titers in the lung, but did manage to clear infection with delayed kinetics, leading to a presumably higher viral exposure over time. Despite this increased viral exposure, they hardly formed iBALT structures in the lung. Conversely, when recombinant IL-1 was administered early after IAV infection to wild-type mice, the formation of iBALT structures was facilitated. Mechanistically, we found impaired CXCL13 chemokine induction early after infection in *Il1r1*^−/−^ mice. Later, at the time when iBALT was fully formed in wild-type mice, we could not detect any differences in CXCL13 levels, suggesting that the instructive signals that condition the lung for clustering adaptive immune cells are given very early (2–4 dpi) after infection. By studying iBALT formation in chimeric mice, we found that IL-1R expression on stromal cells is necessary for proper iBALT formation. The exact cell type of stromal cells that is needed to induce GC B cells, however, still needs to be defined. Whether this stromal cell type is directly responsible for the CXCL13 production needed to initiate iBALT formation or an intermediate cell type is involved remains a matter of debate.

As we observed a decreased induction in MoDCs and these cells are previously described as being major cytokine and chemokine producers (46), it is a possibility that these cells are involved in the CXCL13 induction. Alternatively, LTβ-sufficient B cells can support the progression toward mature, fully structured TLOs (35, 47) most likely *via* a positive feedback loop of CXCL13 production and LTβ expression (24). According to this hypothesis B cells are activated *via* TLR signaling, induce expression of LTβ on their surface, and interact on its turn with LTβR bearing B cells. This LTβ signaling will induce CXCL13 production and release, which attracts more B cells and upregulates LTβ expression. It is an attractive hypothesis that IL-1 might also induce LTβ expression on B cells, although this is hard to reconcile with our observation that a radioresistant cell type responds to IL-1 in our model.

We can only speculate about the source of IL-1α. Previous research has shown that IL-1α can be released by dying cells (48). In this respect, virus-infected lung epithelial cells might be a possible source of IL-1α as it has been observed that IL-1α exerts feedback on epithelial cells and induces a second cytokine and chemokine wave during innate immune responses in the lung (3, 49). We have only measured the mRNA for IL-1β. Secretion of bioactive IL-1β requires activation of the Nlrp3 inflammasome that leads to activation of caspase-1 and cleavage of pro-IL-1β into IL-1β. Infection with IAV leads to activation of the Nlrp3 inflammasome in a process requiring the type I IFN-induced RNAse L/OAS system, while the virus actively suppresses IL-1β production and Nlrp3 activation *via* the NS1 protein (4, 5). Although others have shown that the NLRP3 inflammasome controls severity of infection (9, 11, 50, 51), future studies will have to address if lack of key components of this inflammasome also leads to reduced iBALT formation.

Generally, TLOs are absent in the lungs of healthy adults (52), but bronchus-associated lymphoid tissue can be observed in the lungs of children who are frequently infected by respiratory viruses (53) and in the lungs of adults who suffer from rheumatoid arthritis (28, 54), transplant rejection (55), COPD (56), and IPAH (31). We can only speculate that IL-1 might also be involved in the formation of these TLO structures. IL-1 is certainly a cytokine that has been implicated in the pathogenesis of rheumatoid arthritis, and targeting the IL-1 pathway *via* IL1RA (anakinra) has been used as an alternative biological treatment in patients failing therapy on TNFα blockade. A very common risk factor for rheumatoid arthritis development is smoking, which also leads to COPD. End-stage COPD is also accompanied by TLO formation in the lungs, and these can be sites of production of antibodies to citrullinated antigens, typical of RA patients. In a preclinical model of smoking-induced TLO formation, the production of autoantibodies and TLO structures was reduced in *Il1r1*^−/−^ mice, accompanied by a reduced CXCL13-production in the lungs (57).

During development, neuronal cells give an LTα1β2-independent instructive signal to local fibroblasts to produce CXCL13 and, hereby, attract CD3^−^CD4^+^CD45^+^ LT_i_ cells (58, 59). The crucial step for SLO development is the interaction of LT_i_ cells with stromal lymphoid tissue organizer (LT_o_) cells. This process happens *via* interaction of LTβ expressed on LT_i_ cells and the LTβR expressed on LT_o_ cells. Upon this interaction, LT_o_ cells produce homeostatic chemokines that drive the recruitment of lymphocytes. T cells and DCs are attracted by chemokine CC ligand (CCL)19 and CCL21; B cells are attracted by chemokine CXC ligand (CXCL)13. Expression of vascular cell adhesion molecule (VCAM)1, intercellular adhesion molecule (ICAM)1, and mucosal addressin cell adhesion molecule (MADCAM)1 allow the attracted cells to cluster together. IL-1 has been very well known for its effects of stimulating adhesion molecules on endothelial cells (60). It is tempting to speculate that the effects of IL-1 on radioresistant cells is *via* induction of the crucial adhesion molecules that initially tether a LT_i_-like cell to the circulation and subsequently to initiate a communication between stromal cells and lymphoid cells, that initiates the CXCL13 production.

The LT_i_-potential of T cells was first addressed in a model of thyroid overexpression of CCL21, where it was shown that CD3^+^CD4^+^-activated T cells interacted with DCs at sites of chronic inflammation and subsequently acted as LT_i_ cells in the absence of Id2 activity (37). It has also been suggested that IL-17 signaling is involved during the initiation phase of iBALT formation by inducing CXCL13, but this role for IL-17 remains controversial (34). In two studies on neonatal mice exposed to endotoxin inhalation and on mice with experimental autoimmune encephalomyelitis, respectively, an activated Th17 CD4 T cell population was found to be involved in inducing TLO structures (34, 61). RORC^+^ IL-17-producing cells were also found inside lung TLOs of patients with IPAH. In humans, Th17 cells express the CCR6 receptor, and in the bloodstream of IPAH patients circulating CCR6^+^ cells were fewer, while the ligand CCL20 was produced in the perivascular TLOs (31). However, TLOs seem to develop normally in *Ccr6*^−/−^ mice (34). The induction of TLOs by Th17 cells was dependent on expression of podoplanin, but why this is the case remains unknown. One possibility is that podoplanin is required for retention of Th17 cells at sites of TLO formation (34, 61). The role of Th17 as LT_i_-like cells is still under debate, and it remains to be seen whether all forms of TLO depend on IL-17 production and whether IL-17A and/or IL-17F is involved. In this regard, iBALT induced by infection with modified vaccinia virus Ankara or influenza virus is not affected by deficiency of IL-17A, while *Pseudomonas aeruginosa*-induced iBALT is dependent on IL-17 signaling (35, 62). As IL-17 production by γδ T cells and Th17 cells can be induced by IL-1 (63–66), we also considered the possibility that IL-17 is part of the cascade leading to IL-1α driven iBALT formation. In our hands, IAV infection indeed gave rise to a higher amount of IL-17^+^ CD4 T cells, but treatment with IL-1α 2 dpi could not increase the amount of IL-17^+^ CD4 T cells in the lung, and experiments in which we administered IL-1 to IAV-infected *Il17ra*^−/−^ mice were inconclusive (data not shown). This suggests that, in contrast to SLO formation, the instructive signals can differ depending on the source of initiating antigen or the inflammatory stimulus that is elicited by the used model.

In almost all TLO structures that have been described, the T cell area contained antigen-presenting DCs (17, 31). As DCs activate T cells, it has been suggested that DCs are sufficient for TLO induction (67). This hypothesis is supported by the observation that repeated injection of DCs into the lungs of mice is sufficient for induction of iBALT structures accompanied by induction of myofibroblast differentiation (17, 68). During formation of Peyer’s patches, a CD11c^+^ cell type expressing LTαβ accumulates at the LN anlagen and is necessary for instruction of stromal cells (59). DCs might also directly instruct stromal cells irrespectively of their effects on T cells. In TLO structures induced in the thymus, DCs were specifically necessary for induction of lymph angiogenesis from stromal cells (69), but how DCs induce TLOs is less clear. In virus-induced iBALT, mainly CD11b^+^ DCs or monocyte-derived cells accumulate; these cells express instructive LTα_1_β_2_ while also producing the homeostatic chemokines CXCL13 and CCL19/CCL21 (17). However, in some models, mostly pDCs accumulate, suggesting a functional role for type I IFN. As is the case in TLOs found in end-stage COPD patients and in a murine SLE model (56, 70). Three studies have shown that depletion of DCs leads to disappearance of existing TLO structures, suggesting that DCs are necessary for structural organization and maintenance of TLOs, most likely through trans-presentation of chemokines, or by providing a continuous source of antigen presentation to T cells (16, 17, 69). We did observe reduced numbers of monocyte-derived DCs in *Il1r1*^−/−^ mice, but have not performed experiments in which only DCs lacked IL-1R to study if the effects of IL-1 were cell-intrinsic or resulting from effects of IL-1 on epithelial cells. Indeed, IL-1R triggering on lung epithelial cells is a very well-known trigger for the production of GM-CSF, one of the major cytokines driving activation of monocytes to adopt a DC-like phenotype (49).

In conclusion, we have described a novel role for early IL-1 production in IAV infection to control the formation of iBALT structures *via* induction of CXCL13 in a stromal cell compartment. Future studies will have to address if this effect of one of the best-known proinflammatory and innate cytokines is a general feature of TLO formation at sites of acute and chronic immune stimulation such as infectious disease and autoimmune pathologies and if this can be exploited to induce iBALT formation as part of a mucosal vaccination strategy (71).

## Author Contributions

KN was responsible for conceptualization of mouse experiments, experimentation, data analysis, and preparation of the manuscript. HH and KD provided experimental support for confocal imaging. CG, HH, and BL assisted in conceptualization of experiments and discussion of data and provided feedback for the manuscript. All authors read and approved the final manuscript.

## Conflict of Interest Statement

The authors declare that the research was conducted in the absence of any commercial or financial relationships that could be construed as a potential conflict of interest.
